# Gender and Ethnic Disparities in Teledermatology Clinical Trial Participants: Cross-Sectional Analysis of ClinicalTrials.gov-Registered Trials

**DOI:** 10.2196/46031

**Published:** 2023-06-13

**Authors:** Asghar Shah, Elie Saliba

**Affiliations:** 1 Division of Biology and Medicine Brown University Providence, RI United States; 2 Department of Dermatology Warren Alpert Medical School of Brown University Providence, RI United States

**Keywords:** teledermatology, tele, gender disparities, clinical trial, ClinicalTrials.gov, gender, disparity, disparities, ethnic, dermatology, research subject, participant, skin, derma, derm, dermatologist, dermatology, epidermis

Teledermatology enables the remote viewing of diagnostic images and clinical histories, with the ability to diagnose and offer treatment recommendations [1]. An increase in telemedicine, in the form of teledermatology, for the management of dermatologic diagnoses was observed in early 2020 [2]. This increase in teledermatology coincided with the need for patients to access providers remotely during the COVID-19 pandemic. Despite this increase in teledermatology, the composition of the trial participants underlying the evidence has not been assessed. Given disparities in representation, measured through the ratio of participation in clinical trials to prevalence, across sex, race, and ethnicity, in clinical trials for interventions such as dermatological drugs [3], it is important to assess whether these disparities exist within teledermatology trials.

This analysis evaluates available trial participant data from the ClinicalTrials.gov trial registry of the US National Library of Medicine. We included all studies related to the use of teledermatology. Relevant participant data were extracted for analysis from their respective ClinicalTrials.gov registration when available. These variables included trial status, funding source, and trial participant demographic information.

Our search yielded 35 clinical trials, of which 25 (71%) were completed and 3 (9%) had available results. Of the 35 clinical trials, 7 (20%) are currently recruiting, 2 (6%) have unknown status, and 1 (3%) was withdrawn, with a total mean start year of 2016.62 (SD 4.21). About 77% (27/35) were funded by non–National Institutes of Health, non–US federal funding (ie, industry). In terms of study type, 8 (23%) studies were observational and 27 (77%) were interventional.

Among the completed trials with results [4-6], 1153 participants were analyzed. Women and Hispanic or Latino participants comprised a minority of participants (Table 1): 227 (19.7%) and 114 (9.9%), respectively. The pooled mean age of the participants was 59.12 (14.31) years. While all 3 trials reported gender and ethnicity (Hispanic or Latino status), only 1 reported on the racial composition of the participants. The racial composition among the 296 participants analyzed was as follows: 187 (63.2%) were White, 70 (23.6%) belonged to more than one race, 19 (6.4%) were Asian, 8 (2.7%) were Black or African American, 5 (1.7%) were American Indian or Alaska Native, and 5 (1.7%) were Native Hawaiian or other Pacific Islander. In 2 (0.7%) cases, race was unknown or not reported.

We found 3 completed clinical trials related to teledermatology with results on the registry [4-6]. Pooled counts and proportions indicate an underrepresentation of women and Hispanic or Latino participants. The pooled participant age was akin to that of an older adult (59.12 years). Only one of these studies reported race. Despite contributing to 19% of the US population in 2021 [7], Hispanic individuals comprised only 9.9% of the participants in these trials and are thus underrepresented as a proportion of the population. A study of teledermatology use in the United States reported an elevated proportion of teledermatology use compared to in-person use among female, younger, non-White, and out-of-state patients [8]. In this cohort, female patients comprised 65,023 (65.9%), non-White patients comprised 8920 (15%), and patients aged less than 40 years comprised 62,695 (83.2%) of those using teledermatology. The findings related to teledermatology use differ from the trial enrollment data for the trials we analyzed in this study.

Teledermatology is postulated to improve health equity by eliminating barriers to care. To this end, a retrospective study found a significant reduction in no-show rates among teledermatology visits compared to clinic visits among females, racial/ethnic minority patients, and Medicare/Medicaid beneficiaries [9]. These findings suggest that access to care, measured through no-show rates, may be improved through teledermatology.

None of the 3 studies with results report income or Medicaid beneficiary status. A study of direct-to-patient teledermatology in a low-income, older adult population found that among those older than 65 years, nearly 32.7% of encounters involved use of video and/or photo sharing compared to 60.6% among those aged <65 years [10]. The pursuit of health equity through use of teledermatology should address the intersecting dimensions of digital literacy with demographic and socioeconomic status contexts. Given the potential for teledermatology to improve health equity, it is important for these groups to be enrolled in clinical trials assessing teledermatology.

Our findings indicate that disparities exist in the gender and ethnic composition of trial participants for teledermatology along with a lack of adequate reporting of trial participants by race.

**Table 1:** Characteristics of completed teledermatology studies with results on ClinicalTrials.gov.

Trial characteristic	Total	Identifier		
		NCT00488293	NCT02358135	NCT03241589
Gender (female), n (%)	227 (19.7)	9 (2.3)	147 (49.7)	71 (15.2)
Ethnicity (Hispanic or Latino), n (%)	114 (9.9)	2 (0.5)	100 (33.8)	12 (2.6)
Age (years), mean (SD)	59.12 (14.31)	62.3 (14.4)	49.0 (14.0)	62.89 (14.43)
Reports racial composition?	N/A^a^	No	Yes	No
Study type	N/A	Interventional	Interventional	Observational

^a^N/A: not applicable.
